# Architecture of a dual biocompatible platform to immobilize genistin: fabrication with physio-chemical and in vitro evaluation

**DOI:** 10.1038/s41598-023-49513-3

**Published:** 2023-12-17

**Authors:** S. Esmaeilneia, R. Amiri Dehkharghani, S. Zamanlui Benisi

**Affiliations:** 1 Department of Chemistry, Central Tehran Branch, Islamic Azad University, Tehran, Iran; 2 Tissue Engineering and Regenerative Medicine Institute, Stem Cell Research Center, Central Tehran Branch, Islamic Azad University, Tehran, Iran

**Keywords:** Chemical biology, Drug discovery, Plant sciences, Materials science, Nanoscience and technology

## Abstract

The design of biocompatible cell culture substrates and electrospun nanofibers can improve cell proliferation and behavior in laboratory conditions for tissue engineering applications in medicine. In this research, genistin was obtained by extracting from soybean meal powder, and then by adding polycaprolactone (PCL), genistin nanocapsules were prepared. For the first time, we used a lipophilic nanophase (encapsulated genistin) coated in a hydrophilic nanophase (gelatin /polyvinyl alcohol) as a dual nanosystem by the electrospinning method. In the approach, the nanofibers mimic the natural extracellular matrix, interact favorably with cells being cultured from one side, and raise the local concentration of a bioactive compound at the cell surface. The encapsulated drug which was inserted in fibers with a loading percentage of 92.01% showed appropriate and significant controlled release using high-performance liquid chromatography (HPLC). To prove the experiments, analysis using an ultraviolet–visible spectrometer (UV–Vis), ^1^H NMR spectrometer, Fourier transforms infrared spectrometer (FTIR), mechanical test, scanning electron microscope (SEM) and microscope transmission electron microscopy (TEM) was performed. The sample synthesized with 40% drug using the MTT method exhibited remarkable biological effects, viability, and non-toxicity. Additionally, significant proliferation and adhesion on the mouse fibroblast cell line L929 were observed within a 72-h timeframe.

## Introduction

Due to the many successes in the field of various biocompatible drugs, finding alternatives that have less harmful effects for treatment is not far away^1,2^. Using nanotechnology for drug delivery is one of the methods that can soon be approved for harmless drugs. With electrospun nanofibers, in addition to controlling the amount of drug released, toxicity and damage to healthy cells can be prevented. High surface area, long-term drug release, high encapsulation efficiency, biocompatibility, and affordability are the characteristics of this technology. Recently, there has been a notable interest in using electrospun fibers infused with essential oils or plant extracts for wound healing. These scaffolds have demonstrated the ability to enhance wettability, exhibit biocompatibility, expedite wound healing, and possess augmented antimicrobial properties. The presumed mechanism by which herbal components contribute to the wound healing process is believed to involve the stimulation of fibroblast migration towards the site of injury, in addition to potential antimicrobial effects^3^. In this connection, genistin is one of the natural ingredients that is obtained through the metabolites of leguminous plants, including soybean^4^. Genistin is a glycoside of isoflavone that exerts estrogen-like functions in cells, so it can bind to estrogen receptors and act as an agonist or antagonist. Consequently, it can be used medicinally as a dietary supplement for hormone therapy^5^. Furthermore, there are several biological effects of genistin, such as antioxidant^6^, anti-inflammatory^7^, antibacterial and antiviral activities, angiogenic and anti-metastasis^8^. The effects of genistin as a reducer heparan sulfate accumulation in human mucolipidosis^9^, inhibition of tyrosine kinase in cancer^10^, and protection of the skin exposed to UV light from nitrosative damage^11^ have been reported. Drug delivery systems have been developed to improve the medicinal and therapeutic properties that often contain the medicine in a reservoir. In recent years, considerable attention has been paid to biodegradable polymer nanoparticles as suitable systems for drug delivery. Polycaprolactone is a biodegradable polymer used as a carrier for drug delivery systems^12,13^. So, the study of PCL has been conducted to explore its potential for the advancement of various medical devices, tissue engineering, and biocompatible carriers^14^. Also, polyvinyl alcohol (PVA) and gelatin (GE) are compatible materials that have been used in tissue engineering applications^15,16^. Polyvinyl alcohol-based electrospun nanofiber scaffolds have been used in bone, cartilage, skin, vascular, nerve, and corneal biomedicine^17^. Gelatin is a natural polymer with known wound-healing abilities and a biological propensity to stimulate cell proliferation^18^. Biocompatible nanomaterials convert near-infrared radiation into heat and stimulate hemoglobin to release oxygen in situ for accelerating wound healing^19,20^. On the other hand, adding gelatin to other biodegradable polymers in making nanofibers can improve the mechanical properties, homogeneous morphology, and diameter of nanofibers^21^. New methods using ultrathin multi-component nanofibers have been presented for the rapid development of biocompatible nano drug delivery systems, good drug entrapment efficiency, controlled release, local delivery, and clinically easy^22,23^. Immobilizing nanoparticles (NPs) onto a surface to increase the effective surface area is one method to improve the interaction between the nanofibers' surface and the cultured cells^24^. It was shown that cells over the nanofibers might reveal more area for nanoparticles to adhere and to be internalized^25^.

Hydrophilic nanofibers of gelatin/polyvinyl alcohol prepared by electrospinning process due to their similarity to the cellular environment can promote cell adhesion, proliferation, migration, and differentiation. Additionally, they increase the cell surface's local concentration of certain bioactive genistin. Also, the production of hydrophobic nano-carrier from polycaprolactone allows the effective delivery to create incredibly versatile bioactive drug release mechanisms. Biocompatible Nanofibers with immobilized nanocapsules as a binary system were assessed in terms of chemical structure, morphology, and mechanical properties. Also, the laboratory behavior of the product, cytotoxicity, and cell proliferation in laboratory conditions was evaluated.

## Discussion and results

**Figure 1:**
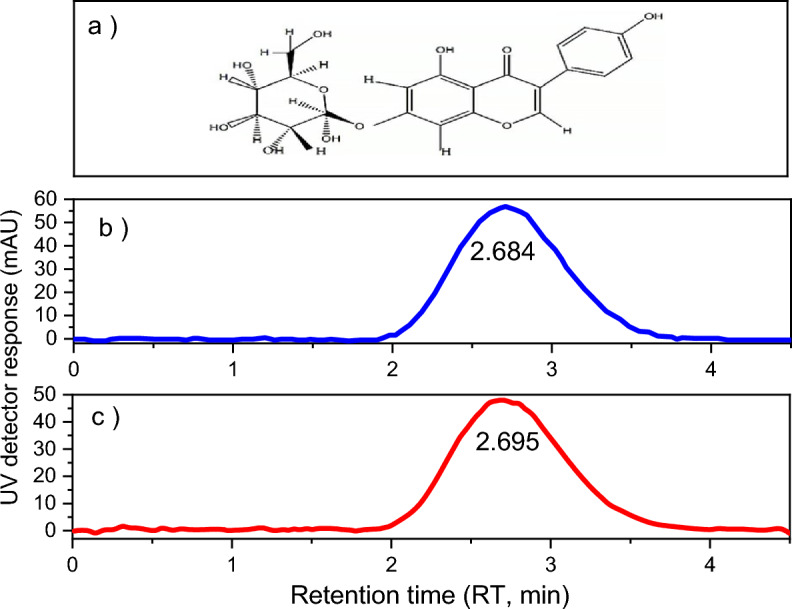
The molecular structure of genistin (**a**), HPLC chromatogram of the standard sample (**b**), and genistin herbal extract (**c**).

In analytical chemistry, HPLC is used to determine and identify the quantity of each component and to separate the members of a mixture. HPLC efficiency is determined by the column's ability to separate the components of a sample by the number of theoretical plates. The practical application of the HPLC method was evaluated through the comparison of standard genistin (Fig. 1b) with genistin herbal extract (Fig. 1c). As can be seen, the herbal extract is a pure substance, and it is the glycoside of genistein (Fig. 1a), which is approved by the retention time of the standard sample (genistin).

**Figure 2:**
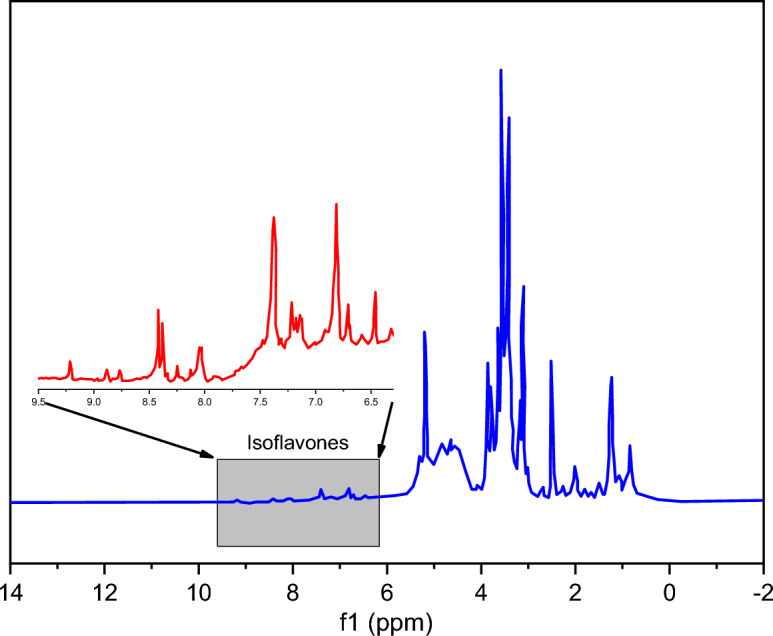
^1^H NMR spectrum of nano-encapsulated genistin in DMSO solvent.

^1^H NMR of nano-encapsulated genistin in DMSO has been shown in (Fig. 2) to confirm the presence of this compound in nanocapsules. As it is clear from ^1^H NMR, genistin, due to having an isoflavone structure, its aromatic protons have appeared in the chemical shift of 6.5–8.5 ppm. But usually, in these compounds, aromatic protons show themselves with a weaker intensity, which is related to the lack of good solubility of this part^26^. Also, the vinyl proton or protons attached to the electronegative functional groups in other materials that were used in making nanocapsules (PCL, span 80, tween 80, or sunflower oil) are placed in the range of δ = 4–6 ppm. The rest of the aliphatic protons of the components of nanocapsules appear in the range of δ = 2–4 ppm^27^.

**Figure 3:**
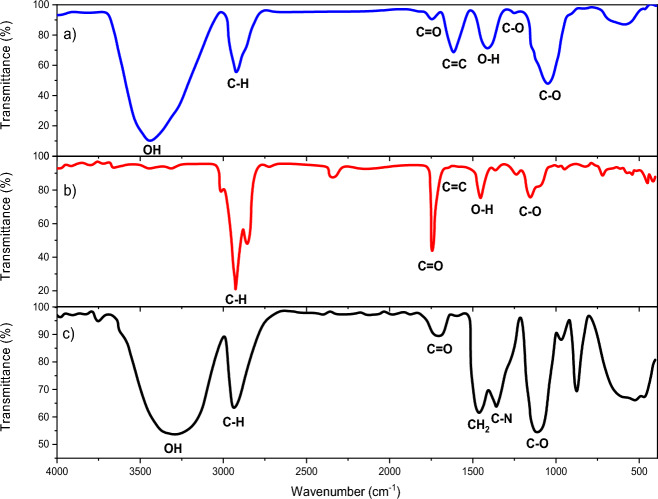
FTIR spectra of (**a**) genistin herbal extract, (**b**) nano-encapsulated genistin, (**c**) electrospun nanofibers.

By comparing the Fourier transform infrared spectrum of the genistin herbal extract in (Fig. 3a) and the nano-encapsulated sample in (Fig. 3b), we realize that the stretching vibration of the –OH functional group has been removed, which can be due to the coating, while the intensity of the vibrational peak of the –C=O group has increased. The finding is related to the presence of this functional group in the polycaprolactone layer. Still, the rest of the vibrations are facing a decrease in intensity, which is related to their coating. Also, in (Fig. 3c), the electrospinning of nanofibers with nano-encapsulated genistin shows a broad and robust band in the 3290 cm^−1^ region, which is the result of a robust intermolecular hydrogen bond between the hydroxyl group of PVA and the amide group of gelatin. In contrast, the small band in the region 2917 cm^−1^ is assigned to the characteristic bands of aliphatic CH stretching vibrations. The decrease in the intensity of the C=O stretching vibration band and its shift to a lower wave number in the region of 1690 cm^−1^ indicates the coating of polycaprolactone with gelatin and PVA layers. The vibrational band at the wave number 1425 cm^−1^ is related to -CH_2_ groups in the second coating of gelatin and polyvinyl alcohol. Also, the stretching vibrations of 1081 cm^−1^ and 1324 cm^−1^ are related to C–O and C–N functional groups, respectively^28^.

**Figure 4:**
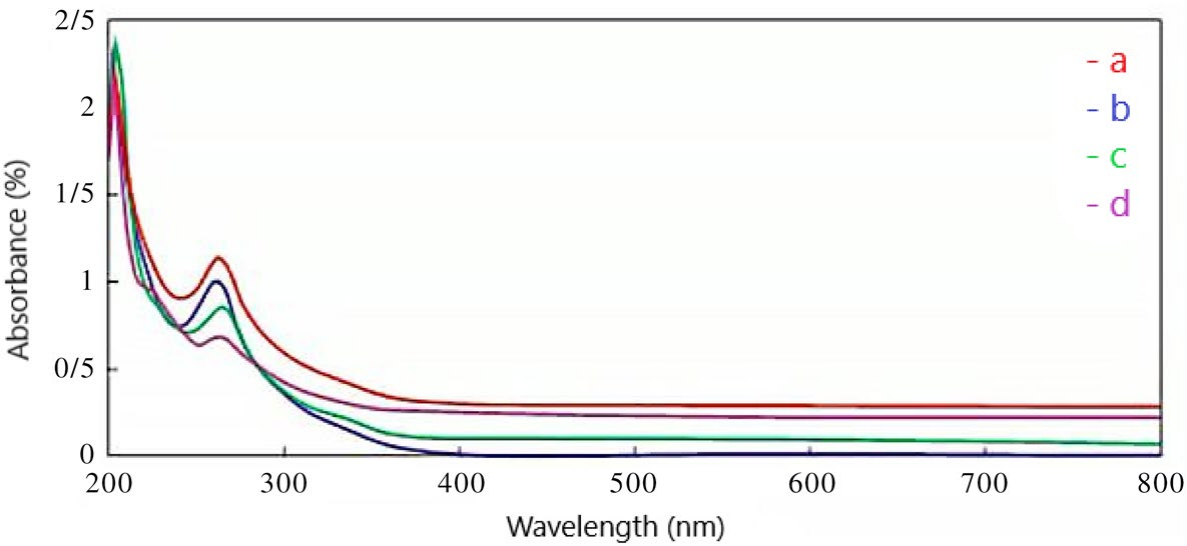
UV–Vis spectra, (**a**) standard genistin, (**b**) genistin herbal extract, (**c**) nano-encapsulated genistin, and d) electrospun nanofibers containing the drug.

The UV–Vis spectra of (a) standard genistin, (b) genistin herbal extract, (c) nano-encapsulated genistin, and (d) the electrospun nanofibers have been shown in (Fig. 4), (0.001 g sample in acetonitrile solvent). Most conjugated unsaturated organic compounds or elements with non-bonding electron pairs absorb energy to electron transfer in the range of 200–800 nm. The absorption wavelength of genistin is 263 nm which can be related to the π → π* transition present in all samples^29^.

**Figure 5:**
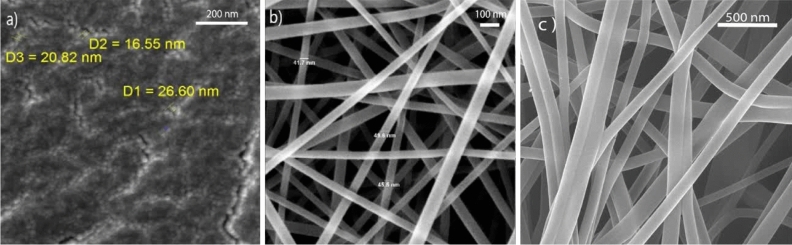
SEM image of (**a**) nano-encapsulated genistin, (**b**) electrospun nanofibers without drug, and (**c**) electrospun nanofibers with drug.

Scanning electron microscope (SEM) collected the reflected electrons from the surface of the irradiated sample to create a visible image of morphology. So, the morphology of nanocapsules containing genistin and electrospun nanofibers has been shown in (Fig. 5a,b). The diameter of nano-encapsulated genistin is 16–26 nm. Also, the size of electrospun nanofibers containing nano-encapsulated drugs varied from 41 to 49 nm. One of the main reasons for using acetic acid to dissolve gelatin is to reduce the surface tension, which leads to a decrease in the diameter of nanofibers^30^, as confirmed by SEM images. The fibers had no beads or structural defects. Since the SEM method was not able to magnify more, therefore, the TEM method was used to visualize the nano-encapsulated drug in the electrospun nanofibers.

**Figure 6:**
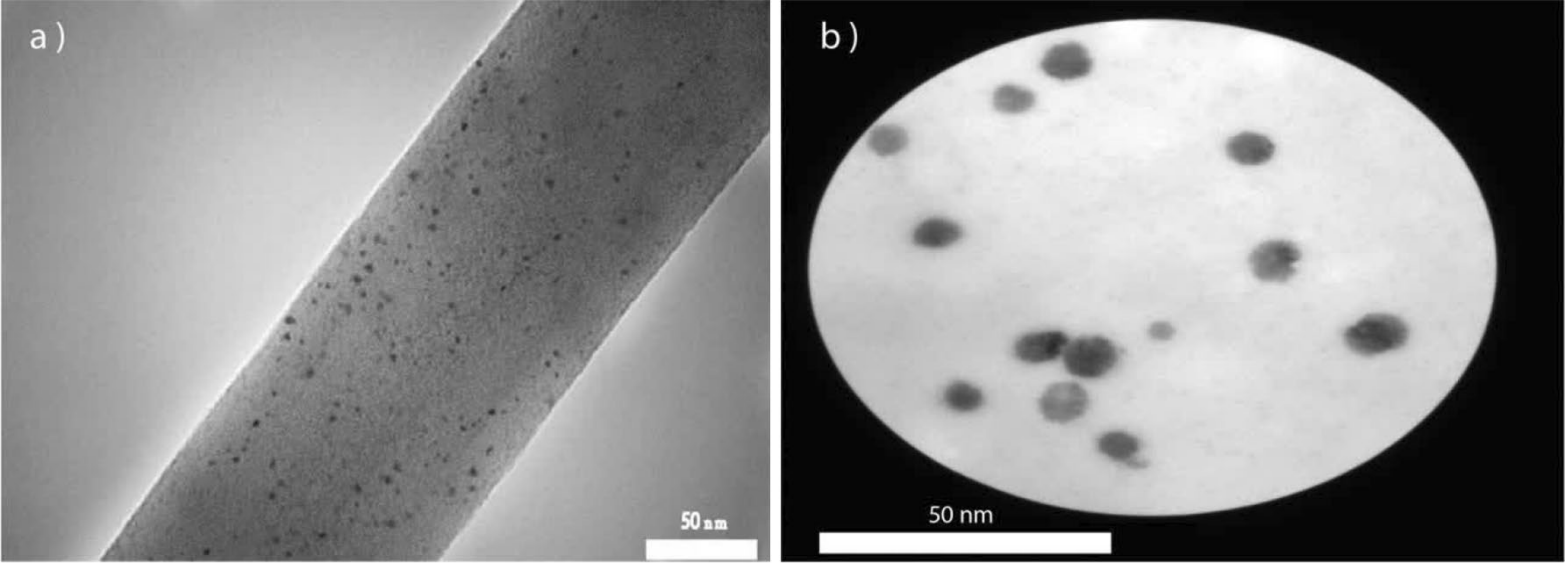
TEM image of electrospun nanofiber with nano-encapsulated genistin (**a**,**b**).

Transmission electron microscopes (TEM) can produce images of nanostructures with higher magnification and resolution than SEM. This technique leads to seeing details with dimensions usually below 20 nm in sample^31^. So, the TEM of electrospun nanofibers (Fig. 6) was used to show the presence of the nano-encapsulated genistin across the fibers. As can be seen in this image, nanocapsules are somehow immobilized inside the electrospun nanofibers, and this achievement guarantees our goal in this work.

One of the essential properties suggested to determine the efficiency of nanofibers is to determine the maximum tensile force that the structure can withstand along the axis. Also, one of the essential parameters evaluated in the tensile test is the amount of pressure in MPa compared to the tension, which expresses the amount of change in the size of the sample compared to its original length in percentage terms. Since PVA is a semi-crystalline structured polymer, it has good chemical resistance and thermo-stability to be a suitable host material. On the other hand, GE fiber can be easily extended into three-dimensional structures to stimulate cell proliferation.

**Figure 7:**
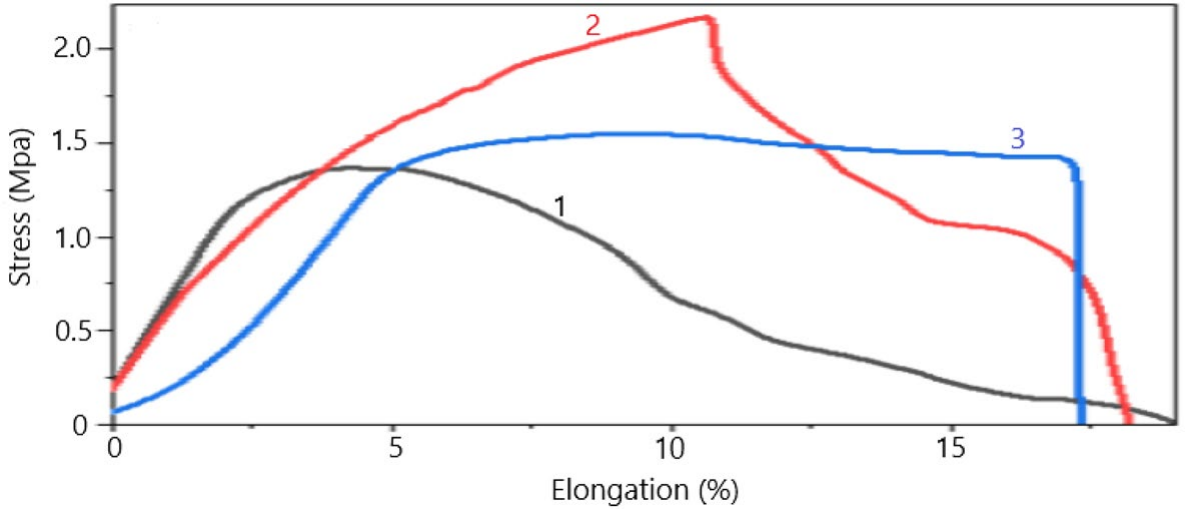
The exploration of the increase in the relative length of electrospun nanofibers as a function of stress, Diagram 1 (with 40% drug), Diagram 2 (with 10% drug), and Diagram 3 (with 20% drug).

**Figure 8:**
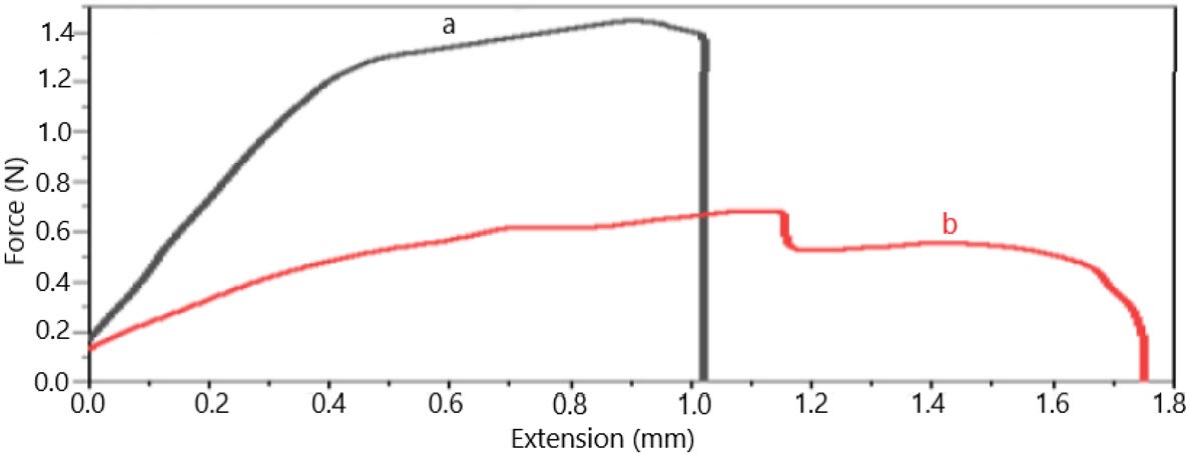
Tensile test in terms of force and extension of (**a**) electrospun nanofibers containing 40% drug, (**b**) electrospun nanofibers without drug.

As a result, mixing PVA with GE leads to structures with high strength due to internal bonds and joints, especially H-bonding. So, adding GE to PVA improves the elasticity and increases the elongation at the breaking point. Also, utilizing methanol to cross-link the PVA/GE mixes resulted in improved mechanical characteristics. The degree of crystallinity was enhanced due to two distinct factors. Firstly, through the application of an alcohol treatment, which effectively eliminated any remaining water content from the fibers, thereby resulting in improvements in both the physical cross-links and mechanical properties^30^. Secondly, by employing semicrystalline polycaprolactone as a nanocarrier for genistin. The elastic modulus for fibers with 40% nano-encapsulated genistin is 45.26 MPa (Table 1), and for fibers without the drug, it is 11.47 Mpa, which indicates that fibers with drug capsules have better resistance. In Fig. 7, diagram 1 shows the mechanical properties of electrospun nanofibers containing 40% nano-encapsulated genistin. Diagram 2 and diagram 3 have 10% and 20% drugs, respectively. According to the results of Table 1, it is observed that while enhancing the quantity of the nanocapsulated drug, the elastic modulus demonstrates an increment. However, a non-linear correlation exists between the concentration of nanocapsules and the elastic modulus. This occurrence can potentially be attributed to the phenomenon of phase separation between the hydrophobic PCL and the hydrophilic PVA/GE, which consequently disrupts the uniformity of the fibers. Furthermore, the strength of electrospun nanofibers with the drug (Fig. 8a) is more than electrospun nanofibers without the drug (Fig. 8b), which is by their tensile strength in (Table 2). The intersection points of the elastic and plastic range in the synthesized nanofibers containing 40% of the nano-encapsulated genistin is 0.78 MPa higher than the electrospun nanofibers without drugs. Also, the drug-free sample has upper and lower yield limits, and its deformation during elongation is non-uniform, indicating inhomogeneity in the structure's strength and undesirable for related applications^32,33^.

**Table 1:** Relative elongation test according to stress.

Items	Elastic module	Break strain (%)	Break extension (mm)	Peak stress (MPa)	Peak force (N)
1	45.26	19.09	3.82	1.39	1.45
2	28.99	18.23	3.64	2.18	0.57
3	43.64	17.20	3.44	1.58	1.64
Delta	–	1.89	0.38	0.79	1.07
Deviation	–	0.77	0.15	0.34	0.47
Mean	–	18.17	3.63	1.72	1.22

**Table 2:** Tensile test according to force and extension.

Items	Break stress (MPa)	Peak stress (MPa)
a	1.35	1.39
b	0.31	0.50
Delta	1.05	0.78
S. deviation	0.52	0.39
Mean	0.83	0.89

**Figure 9:**
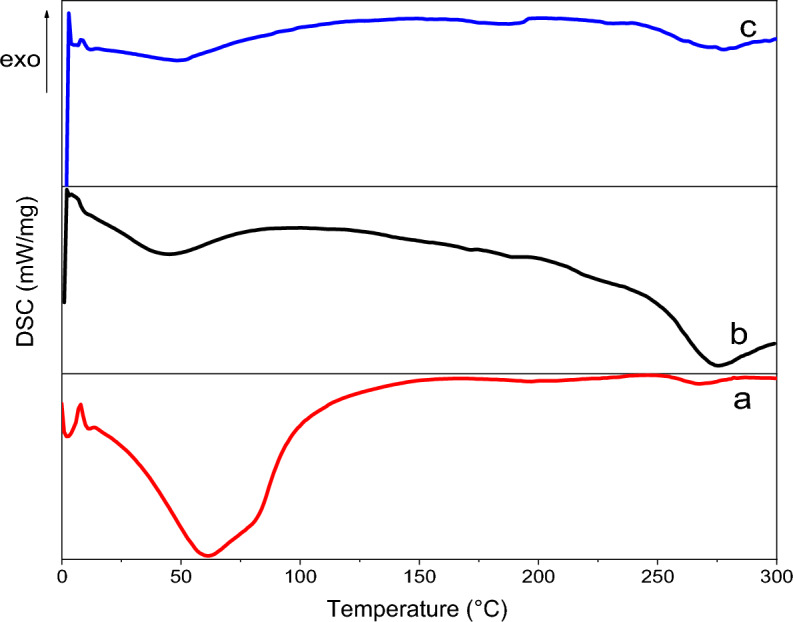
DSC thermograms of (**a**) nano-encapsulated genistin, (**b**) electrospun nanofibers without drug, and (**c**) electrospun nanofibers with drug.

Figure 9 exhibits DSC thermograms of nano-encapsulated genistin (a), electrospun nanofibers without drug (b), and electrospun nanofibers containing 40% nano-encapsulated genistin (c). All thermograms portray two distinct endothermic peaks at approximately 50 °C and 275 °C. Nevertheless, these peaks are associated with different enthalpies, as stated in Table 3. The initial peak occurring at approximately 50 °C, recognized as the dehydration temperature (T_H_), is induced by the existence of water molecules affixed to the considerably hydrophilic groups detected in gelatin, PVA, and especially genistin molecules. The second peak, commonly referred to as the degradation temperature (T_D_), occurs around 275 °C. The elevated T_D_ observed in sample **c** can potentially be attributed to the augmented density of bonds (bonds per volume) resulting from the inter and intra-hydrogenic or electrostatic bonding between the polymeric chains and the genistin. Additionally, the broadening of the peaks in DSC analysis of electrospun nanofibers loaded with the drug confirms a reduction in the presence of unbound hydroxyl (OH) groups^34^. In a general context, the response of the final product to the application of heat is significantly influenced by the influence of semicrystalline PCL and the PVA/GE treated with methanol, which imparts a noteworthy degree of crystallinity.

**Table 3:** The DSC results of nano-encapsulated genistin (a), electrospun nanofibers without drug (b), and electrospun nanofibers with drug (c).

Samples	T_H_ (°C)	∆H_H_ (J/g)	∆H_D_ (J/g)	T_D_ (°C)
**a**	61.7	275.5	1.4	264.2
**b**	43.5	172.0	142.4	273.9
**c**	48.3	96.2	36.2	277.5

**Figure 10:**
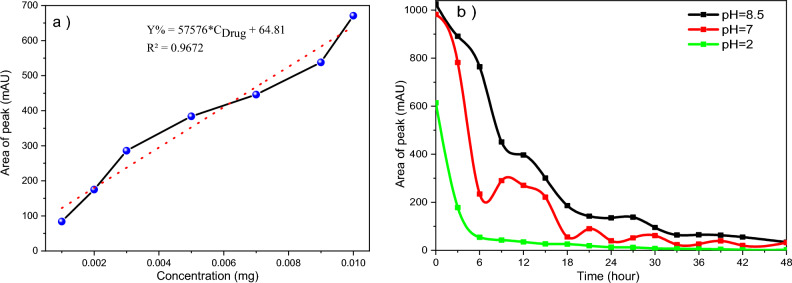
(**a**) Calibration curve of the drug release, (**b**) delivery of genistin at pH 8.5, 7, and 2.

In Fig. 10a, we will examine the calibration of genistin release in electrospun nanofibers containing nanocapsules. The HPLC device checked the sub-peaks at a wavelength of 263 and in 10 min. So, Drug entrapment efficiency (EE% = 92.01% ± 0.7) was calculated using the Eq. (1)^35^.

1 $${\text{EE}} = ({\text{Weight of drug in nanoparticles}})/({\text{Weight of drug fed initially}}) \times 100.$$

The pH serves as a crucial physiological indicator that assumes a pivotal function in upholding the equilibrium of cells and tissues. Moreover, aside from its customary physiological effects, pH additionally contributes to several pathological mechanisms as a stimulus. Consequently, the employment of pH-sensitive nano-delivery systems holds significance in the realm of medical treatment^36^. Figure 10b shows the drug release in environments with various pHs of 8.5, 7, and 2. Since genistin is a polar substance (glycoside of genistein). It tends to diffuse toward a polar environment. Consequently, it has a rapid delivery from PCL to PVA/GE nanofibers and then to the cellular matrix. Drug release in alkaline and neutral environments is slower than in acidic environments, and the pH of the acidic environments, due to the ability to destroy PCL and gelatin, can lead to an explosive release of the active substance. It can be concluded that pH can be effective in drug delivery. In general, a significant release was observed during the first 6 h, and the complete release of the drug lasted for 48 h.

**Figure 11:**
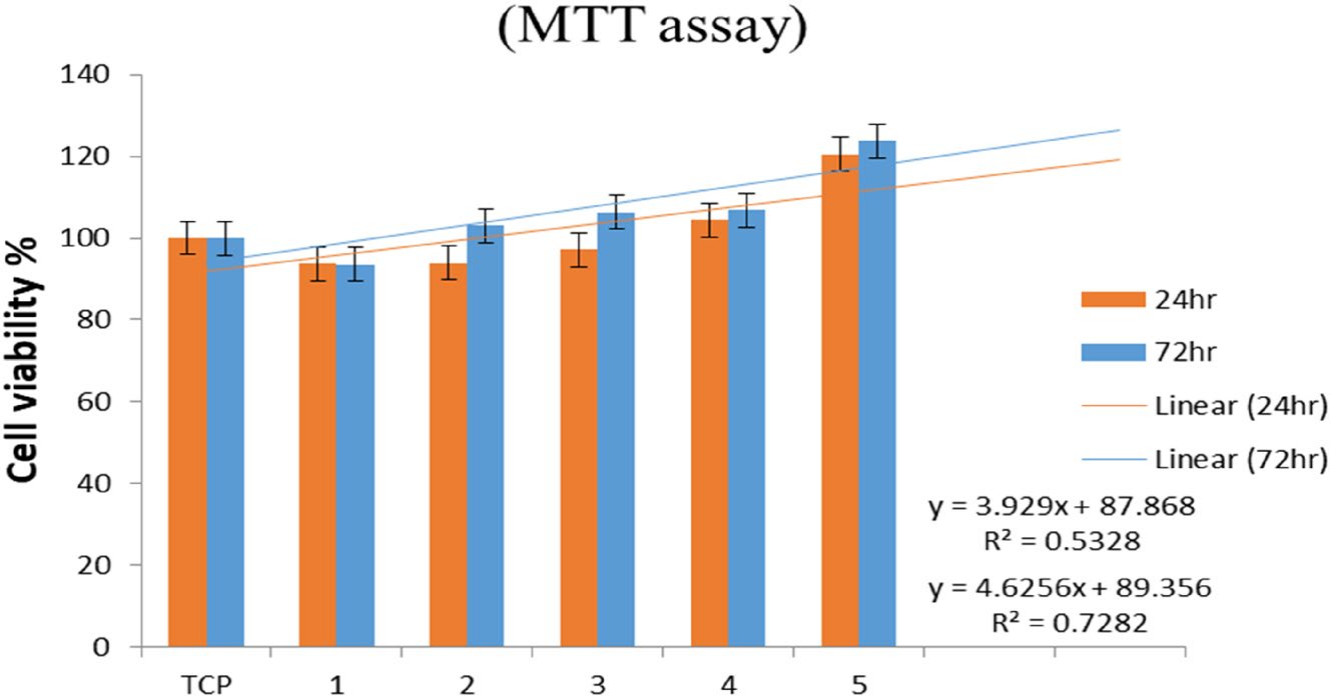
Cell viability of L929 cultured on nanofibers after 24 and 72 h; this test was performed with a significance level of P < 0.01 with ANOVA analysis.

The MTT assay is based on the conversion of 3-(4, 5-dimethylthiazol-2-yl)-2, 5-diphenyltetrazolium bromide (MTT) into formazan crystals by living cells, which determines mitochondrial activity^37^. During the MTT tests on the L929 cell line, the effectiveness of the nanofibers with drug (in different concentrations) and without drugs was compared with the control cell diagram (TCP) by examining the graphs obtained (Fig. 11), and it was well established that the cell viability exposed to electrospun nanofibers containing nano-encapsulated genistin did not show any toxicity at 24 h (red color) and 72 h (blue color). Two factors are effective on cell viability. The first one is the concentration of the drug, and the second one is the electrospinning time (Table 4)^38^. According to the results, sample 5 after 150 min of electrospinning with 40% drug, has the highest cell viability. This phenomenon may be explained by the fact that when the fiber density and amount of drug rise, the likelihood of 3D cell multiplication also increases^39^. But, to understand the biological mechanisms following genistin therapy, it is essential to focus on the potential and innovative pharmacological interventions associated with phytoestrogen-induced wound healing^40^.

**Table 4:** List of examined samples for the MTT test.

Sample	Type of nanofibers	Electrospinning time (min)	Distance (cm)	Flow rate (db)	Voltage (V)
1	Without drugs	90	10	0.6	28
2	With 20% drug	90	10	0.6	28
3	With 20% drug	150	10	0.6	28
4	With 40% drug	90	10	0.6	28
5	With 40% drug	150	10	0.6	28

**Figure 12:**
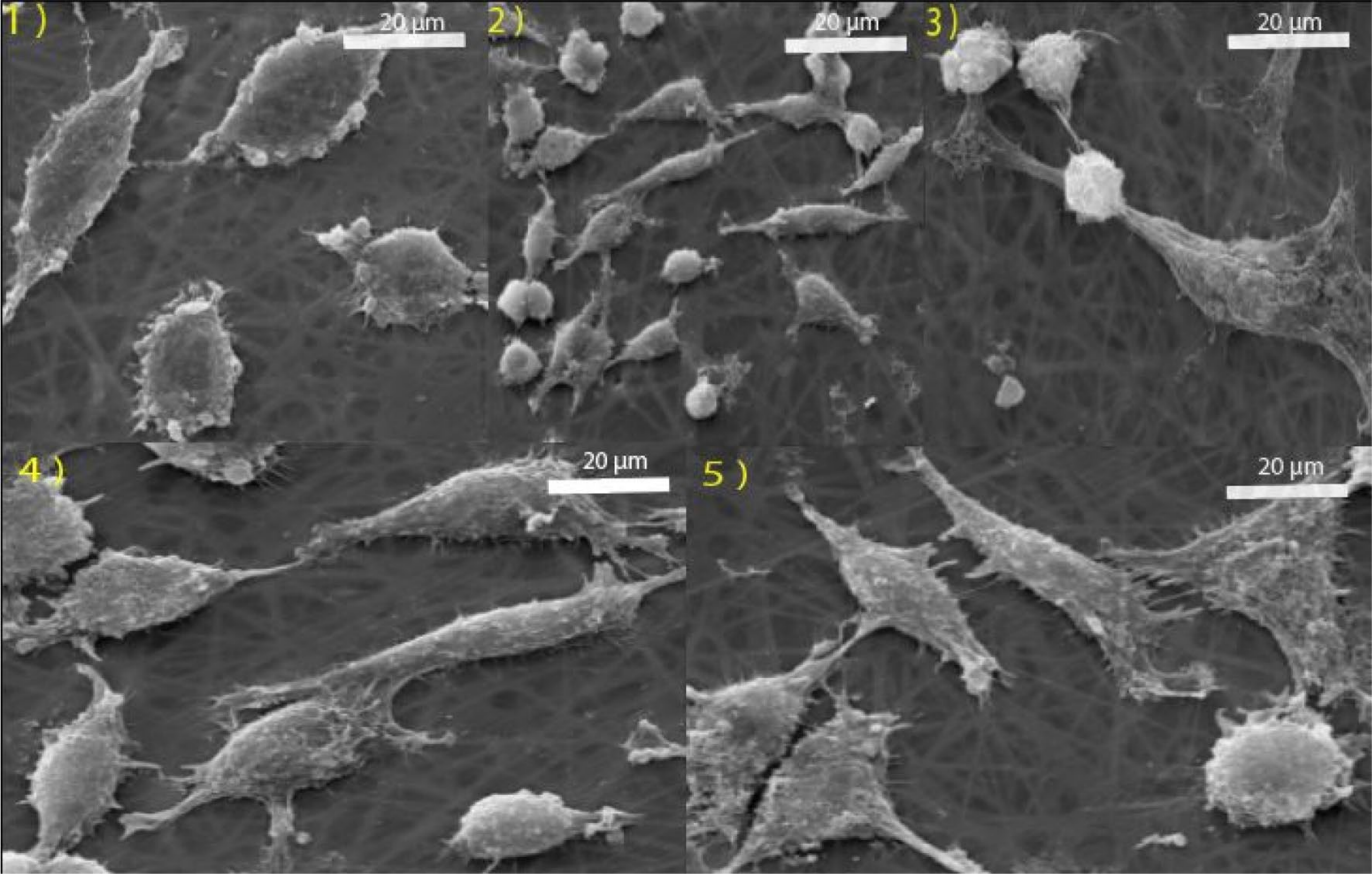
SEM images of L929 cell line proliferation on the nanofibers (samples 1–5).

SEM imaging (Fig. 12) was used to show the morphology of cell adhesion and proliferation of samples 1–5 (Table 3). After 72 h cell culture, samples 2–5 containing the drug have shown more impact than sample 1 (without the drug). The micrograph of the nanofibers seeded with cells showed that the L929 in sample 4 with 40% nano-encapsulated genistin and 150 min electrospinning time is best attached and proliferated on the nanofibers. On one side, the flavonoid structure of genistin and its immobilization in nanofibers make it easily absorbed by L929 cells^41^; on the other side, the hydrophilic two-component PVA/GE provides biochemical signals to promote cell adhesion, migration, proliferation, and differentiation^42^.

## Conclusion

In the project, we tried to select the appropriate constituents for the fabrication of nanofibers that mimic the characteristics of the natural extracellular matrix (ECM). These nanofibers possess the ability to furnish a novel dual nanosystem that facilitates the proliferation and adhesion of L929 mouse fibroblast cells. In this regard, we extracted genistin, one of the most efficacious flavonoids found in soybeans, utilizing a simple one-pot methodology. The validity of the genistin extraction was confirmed through the utilization of HPLC analysis with a standard sample. Subsequently, we employed polycaprolactone, one of the most optimal biodegradable polymers, to encapsulate the aforementioned active ingredient, which was verified via the ^1^H NMR spectrum. The drug loading percentage was determined to be 92.01%. We utilized gelatin and polyvinyl alcohol compounds to fabricate electrospun nanofibers owing to their hydrophilic nature. This approach serves to augment the wettability and hydrophilicity of the nanofibers, thereby facilitating the uniform distribution of cells on the nanofibrous structure. Furthermore, gelatin, a proteinaceous compound, functions as a natural polymer that can promote enhanced adhesion and cell proliferation. On the other hand, we can immobilize nano-encapsulated genistin in an accessible space to facilitate absorption into the cells. For the analysis of the nanofibers containing nano-encapsulated drugs, we employed FT-IR, UV–Vis, SEM, and TEM techniques. The mechanical tensile test corroborated that the nanofibers with a 40% drug content were the outstanding samples. It was found that drug release is slower in alkaline and neutral environments than in acidic environments, with a correlation coefficient of 0.9672. The non-toxic nature of the produced nanofibers was validated by employing the MTT test. PVA/GE nanofibers with nano-encapsulated genistin can easily proliferate L929 cells within a 72-h timeframe. One of the primary obstacles in clinical treatment is the prevention of drug interaction, which has the potential to alter the efficacy of a drug or induce undesirable side effects. To address this issue, a binary biocompatible nano substrate has been proposed as an optimal approach for the nanocarrier of active ingredients, allowing for individual release without any mutual interference, as it is integrated into a scaffold. Furthermore, in the future, alternative dual nanosystems could be developed for sustained delivery by modifying any of the constituent components.

## Materials and methods

### Materials and apparatus

The FT-IR 410, made by Jasco Inc. in Easton, Maryland, USA, used a KBr disc technology to produce spectra with a range of 4000 to 400 cm^−1^. The surfaces of the nanocapsules and the morphology of the nanofibers before and after cell culture were monitored using a Field Emission Scanning Electron Microscope (FE-SEM, SIGMA VP-500, ZEISS business, Germany). Also, the Transmission Electron Microscope (TEM) images were collected by Hitachi (Tokyo, Japan) at 200 kV electron beam energy. Using a UV–Vis spectrophotometer (Perkin Elmer/Lambada 25 UV/Vis spectrophotometer, USA), the product was evaluated between 200 and 400 nm. In the QS 1C ultrasonic bath, the reaction mixture was sonicated. High-performance liquid chromatography (HPLC) was used for the determination of drug loading content and drug delivery. The chromatographic system consisted of a Gemini RP-18 column (150 mm × 4.60 mm, 5 μm, Phenomenex, Torrance, USA) and a Shimadzu instrument (LC-10AVP Pump, UV–Vis SPD-10AVP Module, Class Vp- Software, Shimadzu, Tokyo, Japan). The ^1^H NMR spectra were recorded at room temperature by Bruker AC 400 MHz spectrometers using DMSO-d6 as NMR solvent. The electrospinning apparatus uses a horizontal system with a cylindrical collector covered by aluminum foil (Co881007 NYI, ANSTCO, Iran) at 30 °C. The absorbance Optical Density (OD) was measured on a Microplate reader Labsystems Multiscan, Stat Fax-200. Differential scanning calorimetry (DSC) was acquired using the METTLER TOLEDO KOREA—DSC822e instrument.

The polymer used for this research was poly-ε-caprolactone (Aldrich Chemical, USA) with an average molecular weight of 80,000 Da. Poly (vinyl alcohol) (PVA, 99% hydrolyzed and number average Mw of 31,000–50,000) was obtained from Aldrich Chemical Co (USA). GE Type A (Approx. 300 Bloom, Sigma, St. Louis, MO) was used. All other chemicals and solvents used in the research and synthesis stages were purchased from Merck, Germany. Soybean powder and sunflower oil were purchased from Iranian farms. L929 cells are a murine fibroblast cell line derived from a male C_3_H/An mouse. They are also known as Earles cells, L cells, or NCTC clone 929 cells.

### Preparation of genistin herbal extract

To obtain genistin herbal extract, one gram of soybean meal powder was mixed with ethanol/water (70% and 30%) in an Erlenmeyer flask, and the lid was sealed and placed in an ultrasonic bath. Then we passed it through a filter and put it in a rotary at a temperature of 50–60 °C to evaporate the solvent, and finally, its weight was recorded. The comparison of entirely consistent with the standard sample was performed by HPLC test^43^.

### Genistin extract nano-encapsulation

An adequate amount of PCL/acetone (0.25 g/67 mL) was mixed and covered with foil. Then it was placed in an ultrasonic bath at a temperature of 40 °C. In the second step, we mixed 0.8 mL of sunflower oil (SFO) with 0.196 mL of Span 80 (soluble in water) and added it to the first step with a syringe placed on a magnetic stirrer. Then the genistin (0.00311 g) was added to the previous step under a nitrogen atmosphere and sealed with foil. The organic phase was separated and, after filtration, placed in a vacuum oven to dry. All the encapsulation steps were made once again without herbal extract, and then ^1^H NMR, SEM, FT-IR, and UV–Vis tests were performed, which revealed that the drug was correctly encapsulated^44^.

### Electrospinning nano-encapsulated genistin

According to the optimization reported in Ref.^45^ as a result of employing methanol as a cross-linking agent, at first, a quantity of 0.09 g of polyvinyl alcohol was utilized. Subsequently, 1 mL of double distilled water, heated to a temperature of 90 °C, was added. It was dissolved in a water bath with a temperature of 90 °C at 1400 rpm for 1 h, then 0.09 g of gelatin was mixed with 1 mL of acetic acid in a water bath with a temperature of 40 °C at 1400 rpm for 40 min. Then, polyvinyl and gelatin solutions were cooled to ambient temperature and mixed, afterward, 0.01, 0.02, and 0.04 g of the capsule medicine obtained from the previous steps were added to it, and the resulting substance was poured into a syringe and placed in the electrospinning machine. The optimal value for electrospinning was flow rate (0.6 mL/h), voltage (28 V), nozzle-to-collector distance (10 cm), and the amount is 0.04 g of medicine^46^. To prevent the passage of light, the collector was covered with foil. After that, electrospun nanofibers treated with methanol (soaking in methanol for 8 h) and then was dried in vacuum vacuum-connected desiccator.

### Mechanical tensile test

The electrospun nanofibers were formed with a voltage of 28 (V), a flow rate of 0.6, and a distance of 10 for 4 (h), then the thickness of 0.08, and the dimensions of 5 × 1 cm were prepared and placed inside the aluminum sheet frame, and the models were subjected to a tensile force of 10 newtons at a speed of 2 mm/min until the breaking point, and their tensile properties were calculated.

### Differential scanning calorimetry

Thermal analysis was performed using computerized differential scanning calorimetry (DSC). A DSC thermogram was obtained using a sample weight ranging from 3 to 5 mg and conducted under a controlled nitrogen atmosphere. The experimental procedure involved subjecting the samples to a heating process, starting from 0 °C and gradually increasing up to 300 °C, while maintaining a scanning speed of 10 °C per minute.

### Drug release behavior

The final product was immersed in Buffer solutions with differing pH levels to investigate the quantity of genistin that was administered. Utilizing high-performance liquid chromatography (HPLC), the sample was subjected to analysis at a wavelength of 263 nm using an eluent composed of 85% acetonitrile and 15% water. The analysis was conducted at different pH levels and for varying durations, with a maximum timeframe of 48 h^47,48^.

### MTT assay

MTT dye (with a concentration of 0.005 g/L) was poured on the treated L929 cells with the final sample in the amount of 100 µL and incubated for 4 h at 37 °C. Then the supernatant on the cells was removed, and the formazan crystals were dissolved in 100 µL/well of DMSO. Finally, the concentration of formazan was read with a microplate reader ELX800 at a wavelength of 570 nm. P value < 0.01 was considered statistically significant.

### Cell culture

In order to conduct this investigation, the mouse fibroblast cell (strain C3H/An) was purchased from the Iranian Biological Resource Center (IBRC C10102). L929 was cultured in DMEM (Dulbecco’s Modified Eagle’s Medium), 10% PBS at 37 °C in a humidified atmosphere with 5% CO_2_. Cells were washed twice with PBS for 5 min for subculture and seeding. Electrospun nanofibers were placed on 96 healthy plates and UV sterilized for 20 min. 100 µL of a cell solution (containing 5 × 10^3^ cells) were seeded over the nanofibers and houses without nanofibers (as a control group), and cells were allowed to adhere for 4 h in the incubator. Complete and appropriate media was added to each well, and cells were incubated for up to 24 h and 72 h. After that, the cell culture medium was discarded. Control of cellular adhesion and proliferation was assessed using an inverted light microscope and scanning electron microscopy (SEM) to ascertain cellular compatibility and investigate morphological features^49,50^.

### Morphological study

The surface morphology of nanofibers containing nano-encapsulated genistin before and after cell culture was monitored by scanning electron microscopy (SEM). Cell-seeded scaffolds were washed with PBS and fixed with 2.5% glutaraldehyde for 40 min. Then the scaffolds were dehydrated with increasing alcohol concentration. Finally, the samples were allowed to dry at room temperature (RT) and sprayed with gold. The acceleration voltage of the scanning electron microscope was 24 kV.

### Statistics

The data were statistically analyzed using SPSS 20.0. The data were shown as mean SD. Data analysis for cytotoxicity and biological activity was done using a one-way variance analysis (ANOVA) post hoc LSD test. P ≤ 0.05 was regarded as significant for all tests.
